# Supplementary material for: Priming conditions shape breadth of neutralizing antibody responses to sarbecoviruses
Source: Nat Commun. 2022 Oct 21;13:6285. doi: 10.1038/s41467-022-34038-6 (PMC9586968; doi:10.1038/s41467-022-34038-6)
Supplement: Supplementary file 1 — Supplementary Information [file 41467_2022_34038_MOESM1_ESM.pdf]

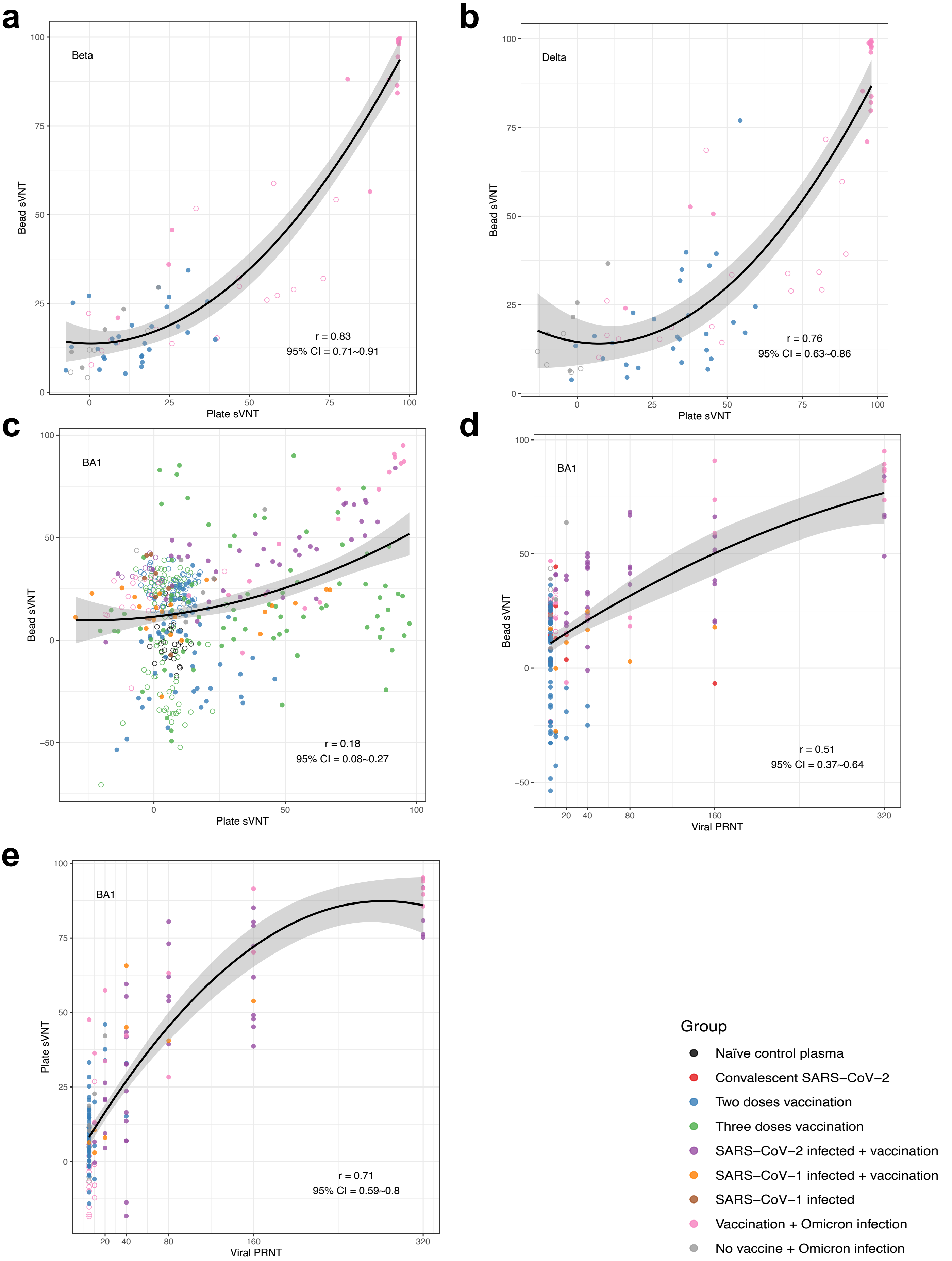

Supplementary Figure 1: sVNT VoC plate versus bead-based multiplex. Spearman correlation analysis (r) analysis of sVNT by commercial plate versus multiplex bead-based assays for SARS-CoV-2 VoC (a) Beta, (b) Delta, and (c) BA.1 Omicron. Omicron results were correlated for PRNT vs bead-based sVNT (d) and plate based sVNT (e). Data represents the mean (bold line), 95% CI (grey shaded), and individual data (open circles for pre/acute samples, closed circles for post vaccination/recovered).
