# Supplementary material for: Priming conditions shape breadth of neutralizing antibody responses to sarbecoviruses
Source: Nat Commun. 2022 Oct 21;13:6285. doi: 10.1038/s41467-022-34038-6 (PMC9586968; doi:10.1038/s41467-022-34038-6)
Supplement: Supplementary file 3 — Description of Additional Supplementary files [file 41467_2022_34038_MOESM3_ESM.pdf]

Description of Additional Supplementary Files

File name: Supplementary Data 1
Description: Subject donor information
