# Supplementary material for: Priming conditions shape breadth of neutralizing antibody responses to sarbecoviruses
Source: Nat Commun. 2022 Oct 21;13:6285. doi: 10.1038/s41467-022-34038-6 (PMC9586968; doi:10.1038/s41467-022-34038-6)
Supplement: Supplementary file 5 — Reporting Summary [file 41467_2022_34038_MOESM5_ESM.pdf]

nature portfolio

Corresponding author(s): Sophie Valkenburg

Last updated by author(s): Jun 30, 2022

Reporting Summary

Nature Portfolio wishes to improve the reproducibility of the work that we publish. This form provides structure for consistency and transparency in reporting. For further information on Nature Portfolio policies, see our Editorial Policies and the Editorial Policy Checklist.

Statistics

For all statistical analyses, confirm that the following items are present in the figure legend, table legend, main text, or Methods section.

n/a	Confirmed	
☐	☒	The exact sample size (*n*) for each experimental group/condition, given as a discrete number and unit of measurement
☐	☒	A statement on whether measurements were taken from distinct samples or whether the same sample was measured repeatedly
☐	☒	The statistical test(s) used AND whether they are one- or two-sided<br>*Only common tests should be described solely by name; describe more complex techniques in the Methods section.*
☐	☒	A description of all covariates tested
☐	☒	A description of any assumptions or corrections, such as tests of normality and adjustment for multiple comparisons
☐	☒	A full description of the statistical parameters including central tendency (e.g. means) or other basic estimates (e.g. regression coefficient) AND variation (e.g. standard deviation) or associated estimates of uncertainty (e.g. confidence intervals)
☐	☒	For null hypothesis testing, the test statistic (e.g. *F*, *t*, *r*) with confidence intervals, effect sizes, degrees of freedom and *P* value noted<br>*Give P values as exact values whenever suitable.*
☒	☐	For Bayesian analysis, information on the choice of priors and Markov chain Monte Carlo settings
☒	☐	For hierarchical and complex designs, identification of the appropriate level for tests and full reporting of outcomes
☒	☐	Estimates of effect sizes (e.g. Cohen's *d*, Pearson's *r*), indicating how they were calculated

Our web collection on statistics for biologists contains articles on many of the points above.

Software and code

Policy information about availability of computer code

Data collection	Multiplex sVNT results were acquired using the MAGPIX system (Luminex, array reader v2.6.1, microplate platform v2.1.15, Bio-Plex manager software v6.2.0.175) following manufacturer's instruction. sVNT plate based ELISA results were quantified using a Wallac MicroBeta JET luminometer 1450 LSC
Data analysis	Software used for data analysis include Prism (v9), Excel, Wallac MicroBeta JET luminometer 1450 LSC for analysis (Promega).

For manuscripts utilizing custom algorithms or software that are central to the research but not yet described in published literature, software must be made available to editors and reviewers. We strongly encourage code deposition in a community repository (e.g. GitHub). See the Nature Portfolio guidelines for submitting code & software for further information.

Data

Policy information about availability of data

All manuscripts must include a data availability statement. This statement should provide the following information, where applicable:

- Accession codes, unique identifiers, or web links for publicly available datasets
- A description of any restrictions on data availability
- For clinical datasets or third party data, please ensure that the statement adheres to our policy

The raw data that support the findings of this study included as supplementary files.

Human research participants

Policy information about studies involving human research participants and Sex and Gender in Research.

Reporting on sex and gender	Sex and gender were not included in our analysis but recorded at recruitment.
Population characteristics	The SARS-CoV-2 infection status of subjects was confirmed by RT-PCR, and prior SARS-CoV-1 infection known. The vaccination status was reported at recruitment at vaccination sites.
Recruitment	Participants were recruited by clinicians during hospitalization of SARS-CoV-2 confirmed infection including mild and asymptomatic cases, or at vaccination clinics across Hong Kong. Participation was voluntary and all provided informed consent.
Ethics oversight	The study was approved by the Joint Chinese University of Hong Kong-New Territories East Cluster Clinical Research Ethics Committee (Ref no: 2020.229), the First Affiliated Hospital of Guangzhou Medical University Ethics Committee (Ref no: 2018.044) and all participants provided written consent. The third dose vaccine study protocol was approved by the Institutional Review Board of the University of Hong Kong (Ref: UW 21-492), and the Clinicaltrials.gov registration number is NCT05057169.

Note that full information on the approval of the study protocol must also be provided in the manuscript.

Field-specific reporting

Please select the one below that is the best fit for your research. If you are not sure, read the appropriate sections before making your selection.

- [x] Life sciences
- [ ] Behavioural & social sciences
- [ ] Ecological, evolutionary & environmental sciences

For a reference copy of the document with all sections, see nature.com/documents/nr-reporting-summary-flat.pdf

Life sciences study design

All studies must disclose on these points even when the disclosure is negative.

Sample size	SARS-CoV-2 convalescent samples obtained from individuals with infection occurring in the period January to March 2020 when the ancestral SARS-CoV-2 was circulating were used, with samples collected at day 30-60 (n=20), day 80-270 (n=20), day 365 (n=22) post infection, with no further vaccination or infection during sampling. This corresponds to the SARS-CoV-2 used in the vaccine, RBD panel and PRNT assay.<br>To assess vaccine immunogenicity, plasma was collected from previously uninfected subjects, prior to receiving the first vaccine dose and at 1 month post 2-dose vaccination with BNTb162b2 or Coronavac (n=30). To assess the impact of prior infection (hybrid immunity), subjects (n=20) who recovered from SARS-CoV-2 infection (346+/-105 days between SARS-CoV-2 infection and vaccination), were assessed 1 month after 1 dose vaccination with BNT162b2 or Coronavac. Participants with prior exposure to SARS-CoV-1 in 2003 were recruited in Hong Kong (HK) and sampled pre- and 1 month post 2-dose vaccination with BNT162b2 (n=7) or Coronavac (n=2). In addition, serum from SARS-CoV-1 infected patients in Guangzhou (GZ), with 'baseline' serum from 2018 (n=10) and post 2-dose vaccination with Sinopharm were sampled at 1 month (n=6), 3 month (n=5) and 6 months (n=2) post vaccination. To assess the impact of heterologous third dose vaccination for either BNT162b2 or Coronavac, individuals were randomized 3 months after 2-doses of vaccination with either Coronavac or BNT162b2 to receive a third dose of Coronavac (CCC) or BNT162b2 (BBB), and samples collected at pre-third dose and 1 month post third dose vaccination (group and timepoint n=20 each). Omicron infection (BA.2 predominant strain at time of serum collection in Hong Kong January-February 2022) of participants (50+/-17 years of age) with prior vaccination of BNT162b2 (n=10), Coronavac (n=7) (some donors are 1, 2 or 3 dose vaccinated), unvaccinated (n= 5) with paired acute (day 0-5) and recovered (1-2 months after illness) samples were tested.
Data exclusions	No data was excluded
Replication	Experiments were repeated twice.
Randomization	Samples were not randomized.
Blinding	Samples were not blinded.

Reporting for specific materials, systems and methods

We require information from authors about some types of materials, experimental systems and methods used in many studies. Here, indicate whether each material, system or method listed is relevant to your study. If you are not sure if a list item applies to your research, read the appropriate section before selecting a response.

Materials & experimental systems

n/a	Involved in the study	
x		Antibodies
	x	Eukaryotic cell lines
x		Palaeontology and archaeology
x		Animals and other organisms
x		Clinical data
x		Dual use research of concern

Methods

n/a	Involved in the study	
x		ChIP-seq
x		Flow cytometry
x		MRI-based neuroimaging

Eukaryotic cell lines

Policy information about cell lines and Sex and Gender in Research

Cell line source(s)	HEK293T and Vero-E6 cell from ATCC
Authentication	Morphology check by microscope, only cells at early passage have been used.
Mycoplasma contamination	In-house mycoplasma testing was performed.
Commonly misidentified lines (See ICLAC register)	none
